# SIRT6 Is Required for Normal Retinal Function

**DOI:** 10.1371/journal.pone.0098831

**Published:** 2014-06-04

**Authors:** Dafne M. Silberman, Kenneth Ross, Pablo H. Sande, Shunsuke Kubota, Sridhar Ramaswamy, Rajendra S. Apte, Raul Mostoslavsky

**Affiliations:** 1 Laboratory of Retinal Neurochemistry and Experimental Ophthalmology, Department of Human Biochemistry, School of Medicine, CEFyBO-CONICET-UBA, Buenos Aires, Argentina; 2 Massachusetts General Hospital Cancer Center, Harvard Medical School, Boston, Massachusetts, United States of America; 3 Department of Ophthalmology and Visual Sciences and Developmental Biology, Washington University, St Louis, Missouri, United States of America; Boston University, United States of America

## Abstract

The retina is one of the major energy consuming tissues within the body. In this context, synaptic transmission between light-excited rod and cone photoreceptors and downstream ON-bipolar neurons is a highly demanding energy consuming process. Sirtuin 6 (SIRT6), a NAD-dependent deacylase, plays a key role in regulating glucose metabolism. In this study, we demonstrate that SIRT6 is highly expressed in the retina, controlling levels of histone H3K9 and H3K56 acetylation. Notably, despite apparent normal histology, SIRT6 deficiency caused major retinal transmission defects concomitant to changes in expression of glycolytic genes and glutamate receptors, as well as elevated levels of apoptosis in inner retina cells. Our results identify SIRT6 as a critical modulator of retinal function, likely through its effects on chromatin.

## Introduction

The mammalian retina is a highly metabolically active tissue and one of the most energy-consuming ones [1]. It requires constant supply of blood glucose to sustain its function and its energy demand is normally met through the uptake of glucose and oxygen [2], [3]. Glucose movement across the blood–retinal barrier occurs mainly through the glucose transporter 1 (GLUT1) [4], [5] and the need for glucose is evidenced by alterations in electroretinogram (ERG) responses and altered neurotransmitter release observed in hypoglycemic conditions [6], [7], [8]. It has been shown that acute hypoglycemia decreases rod and cone vision, blurs central vision, and produces temporary central scotomas in humans [6], [9]–[11]. The consequence of sustained hypoglycemia on retinal function is less clear.

Among many neuronal cellular events, action potential-mediated neuronal communication is believed to be a major process of energy consumption where energy cost comes mainly from postsynaptic receptor activation [12]. In the brain, most of the synaptic activity is mediated by glutamate, thus, the excitatory glutamatergic system represents the single largest energy user, consuming 50% of ATP in the brain [13].

Photoreceptors convert light stimuli to electric impulses. Retinal ON bipolar cells receive direct glutamatergic input from photoreceptor cells. These cells exclusively express the class III G_0_-coupled type 6 metabotropic glutamate receptor (mGluR6 or Grm6) as their primary postsynaptic glutamate receptor [14], [15]. Activation of mGluR6 initiates an intracellular signaling cascade ultimately leading to closure of cGMP gated cation channels and cell hyperpolarization. Thus, energy requirement and consumption in the retina changes greatly according to neuronal activity [1].

Sirtuins are an evolutionarily conserved family of NAD^+^-dependent deacylases that have been involved in many cellular responses to stress, including chromatin modifications, genomic stability, metabolism, inflammation, cellular senescence and organismal lifespan [16], [17], [18]. In mammals, 7 sirtuin isoforms have been described that differ in their subcellular localization and substrates. It is currently accepted that sirtuins are crucial regulators of energy metabolism, likely through sensing changes in levels of intracellular NAD+. Among the members of this family of proteins, SIRT6 appears to have particular significance in regulating metabolism, DNA repair and lifespan. SIRT6 knockout mice appear normal at birth, but they rapidly develop a degenerative process that includes loss of subcutaneous fat, lymphopenia, osteopenia, and acute onset of hypoglycemia, leading to death in less than one month of age [19]. Recently, Zhong *et al*. demonstrated that the lethal hypoglycemia exhibited by SIRT6 deficient mice is caused by an increased in glucose uptake in muscle and brown adipose tissue [20]. At a molecular level, SIRT6 functions as a histone H3K9 and H3K56 deacetylase to control glucose homeostasis by inhibiting multiple glycolytic genes, including GLUT1, and by co-repressing Hif1α, a critical regulator of nutrient stress responses [20].

Given the importance of glucose availability for retinal function, and the critical role of SIRT6 in modulating glycolysis, we undertook this study to characterize SIRT6 in the mouse retina. We found that SIRT6 is expressed and is active in the mouse retina. SIRT6 deficiency has no effect on retinal structure but KO mice retinas exhibited up-regulation of the glucose transporter GLUT1 and down-regulation of the metabotropic glutamate receptor Grm6, indicating that both neurotransmission and glucose levels in the retina might be regulated by SIRT6. ERG analysis showed that the retina of SIRT6-KO mice was profoundly impaired.

## Methods

All animal procedures were in strict accordance with the ARVO Statement for the Use of Animals in Ophthalmic and Vision Research. The ethic committee of the School of Medicine, University of Buenos Aires (Institutional Committee for the Care and Use of Laboratory Animals, (CICUAL) approved this study.

Unless specified, all experiments were performed with retinas from 20-day-old mice.

### *In situ* hybridization (ISH)

ISH for Sirt6 transcripts was performed on paraffin-fixed retinas at the service at the Dana Farber/Harvard Cancer Center (DF/HCC) specialized histopathology research pathology core using RNAscope, according to the manufacturer's instructions.

### Morphologic analysis

Anesthetized mice were intracardiacally perfused with 4% paraformaldehyde as previously described [22]. Eyes were removed, immersed for 24 h in the same fixative, embedded in paraffin and sectioned (6 µm). Sections were stained with hematoxylin and eosin and used for morphometric analysis. Digital images were obtained at 1 mm dorsal and ventral from the optic disk. Thickness of the total retina as well as the individual layers was measured for each eye. Results obtained from four separate sections were averaged and the mean of 4 eyes was recorded as the representative value for each group.

### RNA Isolation

Retinas were homogenized in phenol-guanidine isothiocyanate (Invitrogen Life Technologies). RNA was extracted with chloroform and precipitated with isopropanol by centrifugation (12,000 g) at 4°C. The RNA pellet was washed twice with 75% ethanol and resuspended in 30 µL of water treated with diethylpyrocarbonate. The concentration and quality of total RNA samples were assessed using a NANODROP 2000 spectrophotometer (Thermo Scientific Inc., Newark, DE). Only RNA samples with adequate purity ratios (A260/A280 ¼ 1.9–2.1) were used for subsequent analyses.

### qRT-PCR

For qRT-PCR, total RNA was isolated using RNeasy Mini Kit (QIAGEN) and cDNA was generated using QuantiTect Reverse Transcription Kit (QIAGEN) according to manufacturer instructions. qPCR was carried out using Brilliant SYBR Green qPCR Master Mix Kit (Stratagene). Data were calculated using the ΔCt method. Primers used were as follow: Sirt6-F GGGAACTTGAAGGAACCACA, Sirt6-R AGCCTGGGCTATAGCAGTGA, Glut1-F GCAGTTCGGCTATAACACTGG, Glut1-R GAGACCAAAGCGTGGTGAGT, Grm6-F GTCCATCATGGTCGCCAATGT, Grm6-R AGTCATAGCGTGTGGAGTCAC, β-actin-F ACTATTGGCAACGAGCGGTTC, β-actin-R AAGGAAGGCTGGAAAAGAGCC.

### RNA expression array

Whole retina mRNA from SIRT6 WT and KO mice (n = 3 from each condition) was hybridized onto an Affymetrix Mouse Gene 2.1 ST DNA microarrays. Raw expression values in the form of CEL files were processed and normalized using RMA in the R Bioconductor package (data used in the analysis is available in GEO under accession GSE56563). Clustering of the genes of glutamatergic synapse receptors was performed in R on row-normalized data using the heatmap.2 function with Euclidean distance and the complete agglomeration method. When multiple probe sets were available for a particular gene in a pathway, the probe set with the maximum variance in the data set was chosen.

### Chromatin extraction and Western blot analysis

Western blot analysis was carried out as previously described [21]. The antibodies used were as follows: anti-SIRT6 (1∶1000, Abcam), anti-AcH3K9 (1∶500, Novus Biological), anti-AcH3K56 (1∶500, Sigma), anti-Histone 3 (1∶1000, Abcam), anti- β-actin (1∶5000, Abcam), anti-Grm6 (1∶500, Novus Biologicals), anti-GLUT1 (1∶500, Abcam), horseradish peroxidase-conjugated goat anti-rabbit (1∶2000, catalog # 170-5046; Bio-Rad Laboratories, Hercules, CA, USA or anti-mouse IgG antibody (1∶10000, catalog # 115-035-146; Jackson ImmunoResearch Laboratories, West Grove, PA, USA). Briefly, histone enriched extracts were obtained by homogenizing the retinas in lysis buffer (10 mM Hepes pH 7.4, 10 mM KCl, 0,05% NP-40 plus protease inhibitors (Roche). Homogenates were kept in ice for 20 min and centrifuged at 14.000 rpm for 10 min at 4°C. Supernatant was used for cytoplasmic protein determination and pellets were resuspended in 100 µL of HCl 0,2N. Afer 20 min on ice, samples were centrifuged at 14.000 rpm for 10 min at 4°C. Supernatants were separated and neutralized with 1M TRIS-HCl pH 8. An aliquot was used to determine protein concentration by the Bradford method. Samples (30 µg protein/well) were separated by 4–20% gradient Tris-glycine SDS-PAGE pre casted gel (BioRad). Proteins were transferred to polyvinylidene difluoride (PVDF) membranes, blocked and incubated with the corresponding primary antibodies overnight at 4°C. After washing and incubation for 1 h at room temperature with the indicated secondary antibody they were visualized by enzymatic chemiluminiscence (ECL, Western Blotting Analysis System, Amersham Biosciences, Buenos Aires, Argentina). Developed membranes were scanned and the intensity of bands was determined by using the ImageJ program (National Institutes of Health, Bethesda, MD, USA). Values were expressed as arbitrary units relative to β-actin or total H3.

### Immunoflourescence

After deparaffinization, sections were immersed in 0.1% Triton X-100 in 0.1 M PBS for 10 min. Antigen retrieval was performed by heating at 90°C for 30 minutes in citrate buffer (pH 6.3). Sections were pre-incubated with 5% normal horse serum for 1 h, and incubated overnight at 4°C with the corresponding primary antibodies. After several washings, sections were incubated with the corresponding secondary antibody for 1 h at room temperature (Alexa Fluor 555 Goat Anti-Mouse IgG, 1∶500, Alexa Fluor 488 Goat Anti-rabbit IgG, 1∶500). Regularly, some sections were treated without primary antibodies to confirm specificity. After immunostaining, nuclei were stained with DAPI, mounted with antifade medium and viewed with a fluorescence microscope as described.

### TUNEL

The ApopTag Plus Peroxidase In Situ Apoptosis Detection Kit was use to detect apoptotic cells utilizing terminal deoxynucleotidyl transferase (TdT) specific staining in paraffin embedded retinal sections according to manufacturer instructions.

### Electroretinography (ERG)

Bilateral flash ERG measurements were performed as previously described [23] using a UTAS-E3000 Visual Electrodiagnostic System running EM for Windows (LKC Technologies, Inc., Gaithersburg, MD). Mice (n = 4 for each group) were dark-adapted overnight and anesthetized with 80 mg/kg ketamine and 15 mg/kg xylazine under dim red illumination for electrode placement and testing. Body temperature was maintained at 37±0.5°C with a heating pad controlled by a rectal temperature probe (FHC Inc., Bowdoin, ME). The mouse's head was positioned just inside the opening of the Ganzfeld dome and pupils were dilated with 1.0% atropine sulfate (Bausch & Lomb, Tampa, FL). The recording electrode was a platinum loop 2.0 mm in diameter, positioned in a drop of 1.25% hydroxypropyl methylcellulose (GONAK; Akorn Inc., Buffalo Grove, IL) on the corneal surface of each eye. The reference needle electrode was inserted under the skin at the vertex of the skull. The ground electrode was inserted under the skin of the mouse's back or tail. The stimulus (trial) consisted of a brief, full-field flash (10 µs) either in darkness, or in the presence of dim (30.0 cd/m^2^) background illumination after 10 minutes adaptation time to the background light. The initiation of the flash was taken as time zero. The response was recorded over 250 ms plus 25 ms of pre-trial baseline. Responses from several trials were averaged. The amplitude of the *a*-wave was measured from the average pre-trial baseline to the most negative point of the average trace, and the *b*-wave amplitude from that point to the highest positive point, without subtracting oscillatory potentials. The log light intensity (log [^cd*s^/_m2_]) was calculated based on the manufacturer's calibrations. The amplitudes (in microvolts) of dark-adapted *a*- and *b*-waves and light-adapted *b*-waves were measured from the lowest point of the raw averaged response trace (occurring prior to 50 ms after the flash) to the subsequent highest point (oscillatory potentials were not subtracted).

### Statistical analysis

Quantitative data were expressed as means ± SEM of values obtained from the “n” described in each figure. Significance values were calculated using Student's t-test and differences were considered significant at P<0.05.

## Results

**Figure 1:**
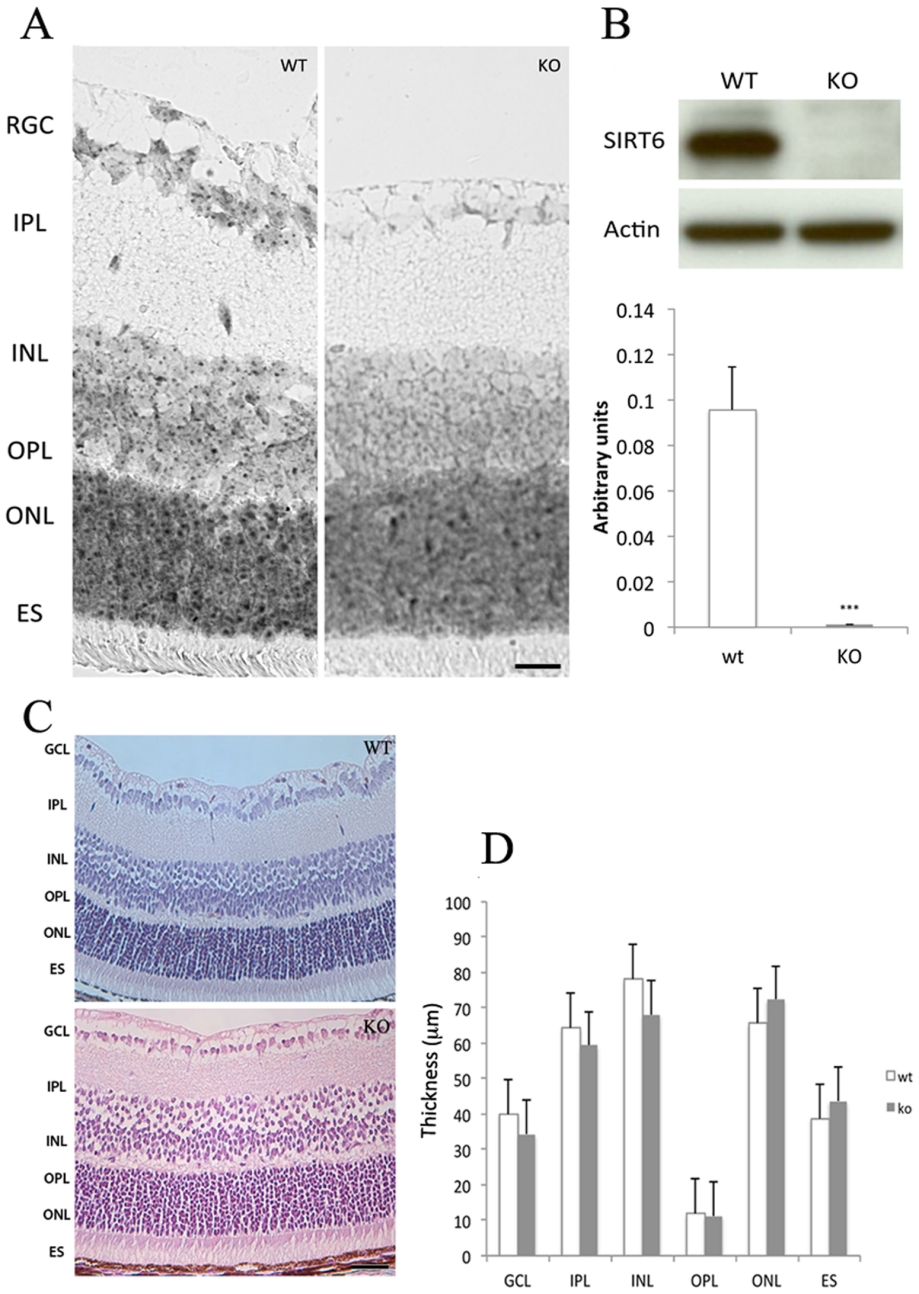
SIRT6 is expressed in the mouse retina. A) Representative *In situ* hybridization showing the expression pattern of SIRT6 in 14-days-old mice (n = 3). Scale bar, 100 µm. B) Chromatin preparations from WT and KO mice retinas were analyzed by Western blot. β-actin was used as loading control (n = 4). Intensity of bands was determined by using the ImageJ software and is represented as arbitrary units. ***p<0.001 C) Retinal histological examination. Representative photographs from H&E stained retinal sections. 20X, scale bar 50 µm from WT and KO. No evident alterations in retinal morphologic features were observed. D) Quantitative analysis of retinal layers thickness. Ganglion Cell Layer (GCL), Inner Plexiform Layer (IPL), Inner nuclear Layer (INL) Outer Plexiform Layer (OPL), Outer Nuclear Layer (ONL), Retinal Pigment Epithelium (RPE). Data are the mean SEM (n = 4). NS, not significant.

In order to characterize SIRT6 in the mouse retina, we first determined the subcellular localization of this sirtuin. The expression of SIRT6 was analyzed by *in situ* hybridization. Characteristic punctuate staining indicative of mRNA expression was observed in the nuclei of cells in all retinal layers of sirt6^+/+^ mice (WT) (Fig. 1A). Consistently, in retina extracts from WT mice abundant protein levels were detected by Western blot, where the specificity of the antibody was confirmed by absence of signal in extract from sirt6^−/−^ (KO) mice (Fig 1B). We then analyzed the structure of the retina in SIRT6 KO mice. The histological analysis revealed no evidence of structure alteration and no difference in the thickness of the whole retina or the individual layers of the KO mice compared to the WT. Moreover, KO retinas showed no evidence of inflammation or infiltrate cells (Fig. 1C–D and Fig. S1). Retinal vasculature was analyzed by a retina fundus image. No alterations were observed and both the number and caliber of vessels showed no difference between WT and KO eyes. Moreover, the underlying pigment was observed to be homogeneous suggesting no obvious retinal pigment epithelial abnormality in these animals (Fig. S1).

**Figure 2:**
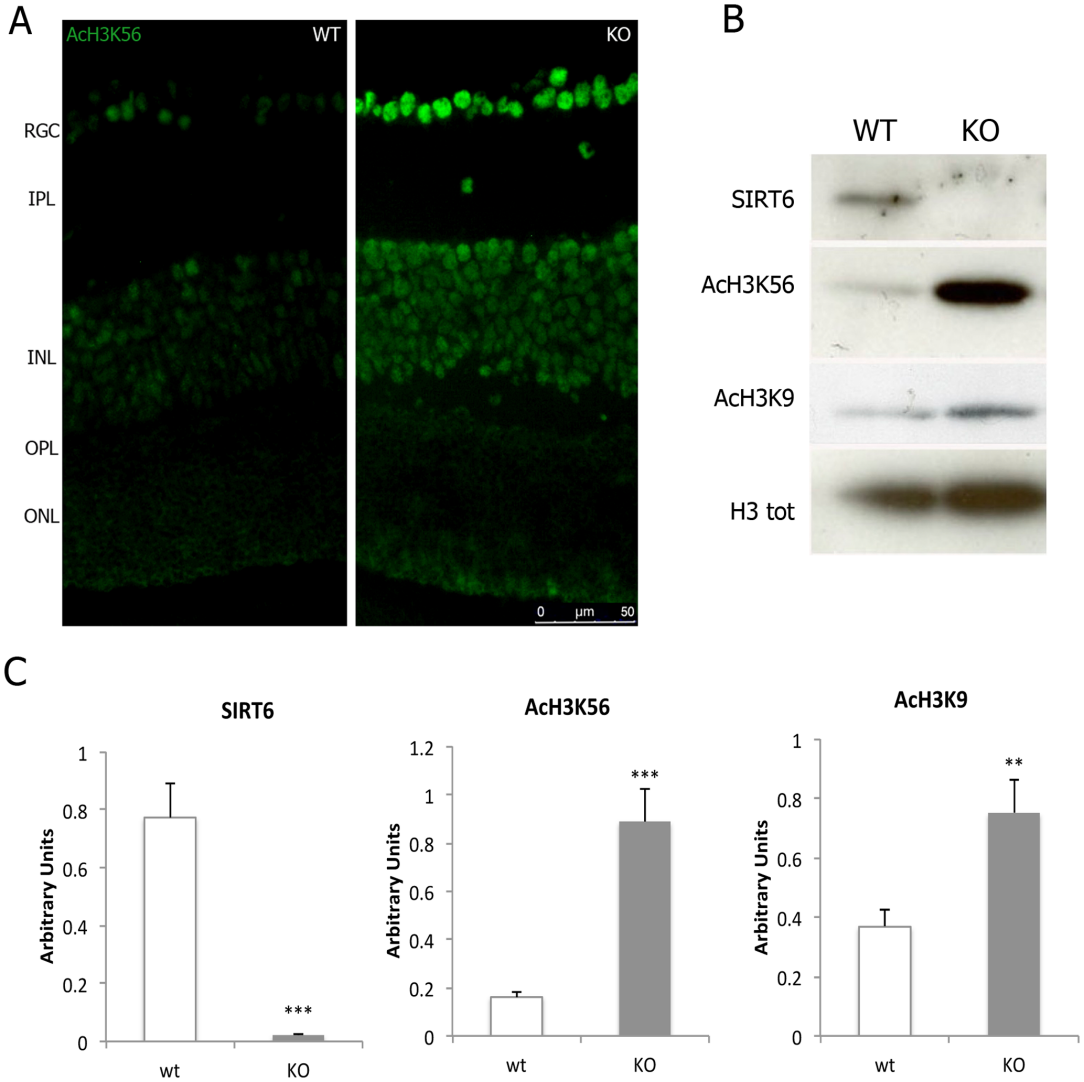
SIRT6 is active in the mouse retina. a) H3K56 acetylation is shown by immunofluoescence. b) Representative Western blot showing protein levels of SIRT6 and the acetylation levels of H3K56 and H3K9 in chromatin preparations from WT and KO mice retinas. Total H3 was used for normalization. c) Quantification of the intensity of bands was determined by using the ImageJ and is represented as arbitrary units. Data are mean ± SE (n = 6 eyes/group). **p<0.01, ***p<0.001

Changes in histone acetylation status can be used as an indication of the level of activity of a histone deacetylase (HDAC). We analyzed the level of acetylation of two of the described substrates of SIRT6: H3K9 and H3K56. Using both immunofluorescence and western blot analysis, we found that both H3K9Ac and H3K56Ac were significantly increased in retinal chromatin preparations from KO mice compared to WT mice, indicating that SIRT6 is active in the mouse retina, and lack of SIRT6 causes a profound change in those chromatin modifications (Fig. 2 and Fig. S2).

**Figure 3:**
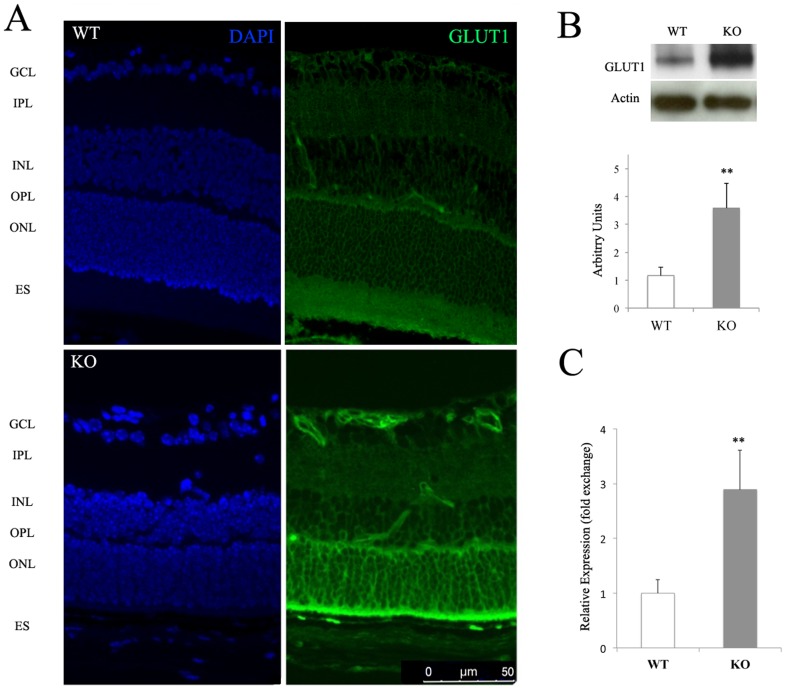
GLUT1 is up-regulated in SIRT6-KO retina. a) GLUTl immunoreactivity in cross-section of WT and SIRT6-KO mice retina. Ganglion Cell Layer (GCL), Inner Plexiform Layer (IPL), Inner nuclear Layer (INL) Outer Plexiform Layer (OPL), Outer Nuclear Layer (ONL), Retinal Pigment Epithelium (RPE). GLUT1 protein (b) and mRNA levels (c) were determined by Western blot and RT-PCR respectively. β-actin was used as loading control. Data are mean ± SE (n = 6 eyes/group) **p<0.01

It has been described that SIRT6 deficiency in mice renders a phenotype characterized by a profound hypoglycemia [19] and that SIRT6-dependent deacetylation of histone H3 at lysines 9 and 56 is required for the regulation of genes associated with glucose metabolism, including GLUT1 [20]. Since this transporter is the main one responsible for the transport of glucose across the blood–retinal barrier (BRB) [4], [5], we determined GLUT1 expression in WT and KO retinas. In accordance with previous findings in other tissues [20], GLUT1 was found to be up-regulated in KO retinas, using immunofluorescence (Fig.3A), immunoblotting (Fig. 3B) and PCR (Fig. 3C), suggesting that glucose availability may be altered in this tissue. Up-regulation of this transporter was evident in all retinal layers and, interestingly, in inner retinal vessels. Levels of another transporter, GLUT4, were not affected (data not shown), suggesting that the effect of SIRT6 is quite specific.

**Figure 4:**
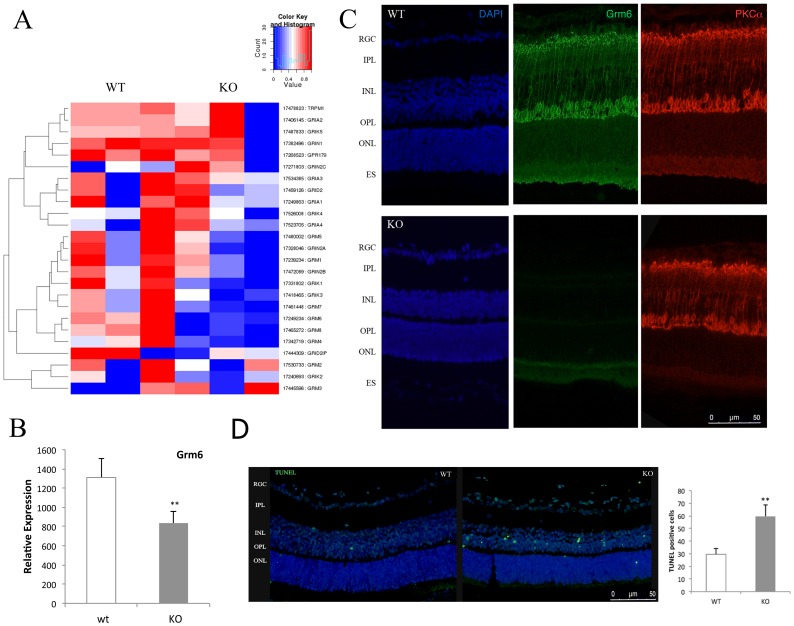
Grm6 is down-regulated in SIRT6-KO retinas. Whole retina mRNA from WT and KO mice was used to profile the expression of several key genes of glutamate receptors involved in the synaptic transmission in an Affymetrix Mouse Gene 2.1 ST DNA microarray. a) Heatmap representing the hierarchical cluster analysis shows the differential expressed mRNAs between WT and SIRT6 KO retinas. The graphic depicts the expression levels of ionotropic AMPA glutamate receptors (Gria1–4), Glutamate receptor, ionotropic kainate (Grik1-2-4-5), Glutamate [NMDA] receptors (Grin1-2a-c) and metabotropic glutamate receptors (Grm1–8). The expression data for the hierarchical clustering image has been row normalized to a range of zero to one with blue representing the row minimum and red representing the row maximum. b) RNA was purified from SIRT6 WT and KO retinas, and Grm6 levels analyzed by RT-PCR. c) immunofluorescence was performed in SIRT6 WT and KO retinas with the indicated antibodies. PKC-alpha was used as a marker for ON bipolar cells. Ganglion Cell Layer (GCL), Inner Plexiform Layer (IPL), Inner nuclear Layer (INL), Outer Plexiform Layer (OPL), Outer Nuclear Layer (ONL), Retinal Pigment Epithelium (RPE). Data are mean ± SE (n = 4) **p<0.01 d) Representative fluorescent images of TUNEL analysis performed in WT and SIRT6 KO retinal sections. Apoptotic nuclei (bright green dots) labeled with fluorescein-dUTP were visualized by fluorescence microscopy. Data are mean ± SE (n  = 3) **p<0.01

In order to understand at the molecular level how SIRT6 acts in the retina, we performed a microarray analysis on total retinal RNA from 15 days-old animals. Among the genes that showed a significant variation between WT and KO animals, several genes involved in glutamatergic synapse (Grm4-8 and the kainate/AMPA-type ionotropic glutamate receptor, GRIK1 and 2) were found to be significantly downregulated in the retinas of KO mice (FIG. 4A). It is worth noting that the most significant difference was found for the metabotropic receptor Grm6, described to be expressed exclusively in ON bipolar cells. This result was confirmed by quantitative PCR and immunostaining (Fig 4B–C). Down-regulation of Grm6 might cause inner retinal neurons to be exposed to a higher amount of endogenous glutamate that might result in severe excitotoxic degeneration [24]. Therefore, we analyzed the presence of apoptotic cells by TUNEL. Notably, a significant increase of dead cells was observed in the inner nuclear layer of SIRT6 KO retinas (Fig 4D).

**Figure 5:**
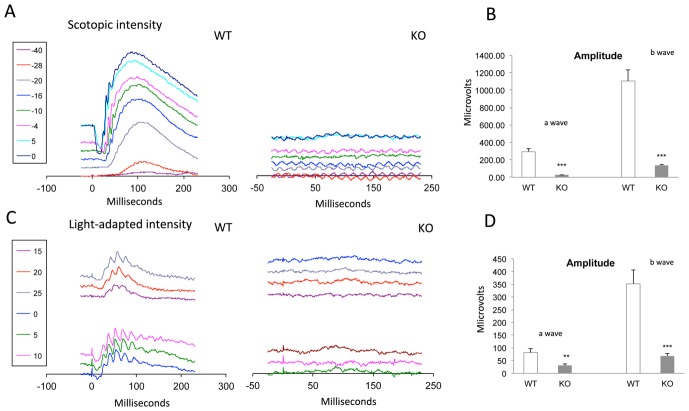
Retinal functional evaluation. Representative scotopic (A) and photopic (C) electroretinograms from WT and SIRT6-KO mice at different light intensities (dBs). Plots B and D depict average amplitudes of *a*-wave and *b*-wave. Note that the fold decrease of the scotopic *a*-wave amplitude (8) is greater than the fold decrease of the photopic *a*-wave amplitude (2,5). Data are mean ± SE (n =  4). **p<0.01, ***p<0.001.

Synaptic transmission is a highly energy demanding process and multiple molecular events occur during synaptic activation, including receptor trafficking, signaling, and metabolic processes. In order to analyze if the changes in gene expression observed would have a functional consequence we assessed retinal function by electroretinography (ERG). As shown in Fig. 5, both scotopic and photopic *a*- and *b*-wave amplitudes were severely altered for all the light intensities analyzed, indicating that absence of SIRT6 profoundly impaired retinal function.

## Discussion

The retina is one of the major energy consuming tissues within the body, maintaining high glucose metabolism for its energetic needs. Moreover, synaptic transmission is a highly energy demanding process, therefore the retina evolved mechanisms to coordinate synaptic activity with glucose homeostasis.

SIRT6 is a member of the sirtuin family of NAD(+)-dependent deacylases that acts to maintain homeostatic control of glucose metabolism by repressing a number of enzymes involved in this process [20]. Although SIRT6 plays a critical role in modulating bioenergy homeostasis and that metabolic control is essential for vision, very little is known regarding this enzyme's involvement in retinal physiology. In this study, we aimed for the first functional characterization of SIRT6 in the mouse retina.

Recent studies have shown that SIRT6 is expressed in the mouse retina and its levels seem to be higher in this tissue compared to brain, heart, liver or kidney [25]. We confirmed the presence of SIRT6 in the mouse retina and were able to determine that it is expressed in all retinal layers. Moreover, SIRT6 was found to be active in the retina since lack of this enzyme resulted in significantly increased level of acetylation of two of its described substrates, H3K9 and H3K56, in KO retinas compared to WT.

Our previous studies demonstrated that SIRT6 deficiency in mice renders a phenotype characterized by a profound hypoglycemia [19] due to increased glucose uptake. Molecularly, SIRT6 deacetylates H3K9 and H3K56, repressing expression of glycolytic genes, including the glucose transporter GLUT1 [20]. In the absence of SIRT6, those genes are up-regulated, causing uncontrolled glucose uptake and a switch towards glycolytic metabolism. Since GLUT1 is the sole transporter responsible for the transport of glucose across the blood–retinal barrier (BRB) [4], [5], we focused on this transporter. Indeed, we found that mRNA and protein levels of GLUT1 were significantly increased in retinas from SIRT6 KO mice, suggesting a glucose imbalance in this tissue. It is expected that GLUT1 upregulation would be accompanied by an increase of glucose availability in the retina making difficult to explain the defects we observed. On the other hand, upregulation of GLUT1 could as well indicate a compensatory mechanism due to local low levels of glucose since, as we published, blood glucose is being mostly uptake by skeletal muscle and adipose tissue in SIRT6 deficient mice [20]. Whether such chromatin-dependent changes in GLUT1 play a causal role in the functional defects we observed in the SIRT6 KO retinas remains to be determined.

Glutamate is the major excitatory neurotransmitter in the central nervous system, and plays important roles in regulating cell excitability and synaptic transmission. Multiple molecular events are coordinated during synaptic activation, including sodium pump activity, receptor trafficking, cytoskeletal rearrangements, signaling, and metabolic processes, making synaptic activity an energetically costly process [26]. Microarray analysis on total retinal RNA revealed that several members of the class III of the metabotropic glutamate receptor family (Grm4–8), and the kainate/AMPA-type ionotropic glutamate receptor (GRIK1–2), are down-regulated in retinas from SIRT6 KO mice. Synaptic transmission between light-excited rod photoreceptors and downstream ON-bipolar neurons is indispensable for dim vision in the mammalian retina. Indeed, disruption of this process leads to congenital stationary night blindness in human patients [27]. Notably, among the metabotropic receptors analyzed, Grm6 was found to be the most significantly down-regulated in KO retinas.

Once released from the pre-synapsis, glutamate can bind to different glutamate receptors on the post-synapsis or can be removed from the synaptic cleft by high affinity glutamate transporters located on adjacent neurons and surrounding glial cells to prevent cell death. The major pathway of glutamate metabolism consists of glutamate uptake by glutamate transporters followed by enzymatic conversion of glutamate to non-toxic glutamine by glutamine synthetase. When a glutamate transporter is pharmacologically blocked, inner retinal neurons are exposed to a higher amount of endogenous glutamate, resulting in severe excitotoxic degeneration [24]. These observations suggest that glutamate is neurotoxic when the uptake system is impaired rather than when the release is excessive. Alternatively, impaired expression of a post-synaptic receptor may contribute to the accumulation of the neurotransmitter in the inter-synaptic space. In this context, down-regulation of Grm6 could account for the increment of glutamate in the inter-synaptic space exerting a toxic effect that could explain the increase of TUNEL positive cells (bipolar cells or Müller cells or both) found in the inner nuclear layer of SIRT6 KO retinas. Since both bipolar and Müller cells are involved in the generation of the *b*-wave, apoptotic cells in the inner nuclear layer would explain the alteration observed in the ERG.

Although no structural differences were observed between WT and KO retinas, functional analysis showed a significantly impaired ERG in KO animals where both *a* and *b* waves were severely compromised. Interestingly, cones would be less affected than rods since the fold decrease of the *a*-wave amplitude in the photopic ERG is less than the fold decrease in the scotopic ERG (2.5 vs. 8). Whether lack of SIRT6 is affecting photoreceptor activity specifically requires further studies. Taken together, up-regulation of GLUT1, down-regulation of Grm6 and the increased number of apoptotic cells in retinas from *sirt6* ^−/−^ mice could account, at least in part, for the visual deficiency observed in these mice.

Overall, our studies demonstrated that SIRT6 deficiency causes major chromatin changes in the retina, which is accompanied by marked changes in expression of metabolic genes and metabotropic receptors, likely explaining the severe functional impairment observed in the SIRT6-KO retinas. Previous studies established sirtuins as critical modulators of metabolism, protecting against metabolic- and age-related diseases, such as diabetes, metabolic syndrome, cancer and neurodegenerative disorders [28]. Our results indicate that SIRT6 plays an important role in maintaining normal retinal function.

Altered methylation patterns and histone modifications have been identified in different ocular diseases like diabetic retinopathy, glaucoma and age-related macular degeneration [29], [30], [31]. However, a comprehensively characterized epigenomic signature for any ocular disease remains elusive. Several questions arise: is there any epigenetic mark that would predict the onset or the progression of an ocular disease? Do epigenetic factors regulate other cellular pathways (other that glucose metabolism and glutamatergic synapse) that may alter visual function? Although epigenetic therapies have proven to be effective in cancer applications [32], the benefits of these approaches have not yet been applied for human ophthalmic diseases.

## Supporting Information

Figure S1 **Histological analysis and eye fundus.** a) Representative low magnification (4X) cross-sections of eyes from WT and SIRT6KO mice. General structure, size and optic nerve head can be observed. No distinctive alteration is detected in SIRT6KO retinas b) Representative fundus images were taken from WT and 2-week-old mouse using the endoscopic fundus imaging system. Posterior pole of the fundus can be observed.
(TIF)

Figure S2 **H3K9 acetylation is shown by immunofluoescence. Ganglion Cell Layer (GCL), Inner Plexiform Layer (IPL), Inner nuclear Layer (INL) Outer Plexiform Layer (OPL), Outer Nuclear Layer (ONL), Retinal Pigment Epithelium (RPE).**
(TIF)

File S1 **Supporting Methodology.**
(DOCX)

## References

[1] Niven JE, Laughlin SB (2008) Energy limitation as a selective pressure on the evolution of sensory systems. J Exp Biol 211: 1792–1804. 18490395 10.1242/jeb.017574

[2] Graymore CN (1970) Biochemistry of the retina. In: Graymore CN, ed. Biochemistry of the Eye. London: Academic Press. pp 645–735.

[3] Winkler BS (1981) Glycolytic and oxidative metabolism in relation to retinal function. J Gen Physiol 77: 667–692. 6267165 10.1085/jgp.77.6.667 PMC2215447

[4] Mantych GS, Hageman GS, Devaskar SU (1993) Characterization of glucose transporter isoforms in the adult and developing human eye. Endocrinology 133: 600–607. 8344201 10.1210/endo.133.2.8344201

[5] Kumagai AK, Glasgow BJ, Pardridge WM (1994) GLUT1 glucose transporter expression in the human diabetic and non-diabetic eye. Invest Ophthalmol Vis Sci 35: 2887–2894. 8188484

[6] Khan MI, Barlow RB, Weinstock RS (2011) Acute hypoglycemia decreases central retinal function in the human eye. Vis. Res 51: 1623–26. 21601590 10.1016/j.visres.2011.05.003 PMC3149775

[7] Skrandies W, Heinrich H (1992) Differential effects of mild hypoglycemia on proximal and distal retinal structures in man as revealed by electroretinography. Neurosci. Lett. 134: , 165–8. 10.1016/0304-3940(92)90507-4 1589141

[8] Umino Y, Everhart D, Solessio E, Cusato K, Pan JC, et al (2006) Hypoglycemia leads to age-related loss of vision. Proc. Natl. Acad. Sci U. S. A. 103: 19541–45. 17159157 10.1073/pnas.0604478104 PMC1697832

[9] McCrimmon RJ, Deary IJ, Huntly BJ, MacLeod KJ, Frier BM (1996) Visual information processing during controlled hypoglycaemia in humans. *Brain* 119: 1277–1287. 8813290 10.1093/brain/119.4.1277

[10] Ewing FM, Deary IJ, McCrimmon RJ, Strachan MW, Frier BM (1998) Effect of acute hypoglycemia on visual information processing in adults with type 1 diabetes mellitus. *Physiol Behav* 64: 653–660. 9817577 10.1016/s0031-9384(98)00120-6

[11] Barlow RB, Farell B, Khan M (2003) Metabolic modulation of visual sensitivity. *Adv Exp Med Biol* 533: 259–267. 15180272 10.1007/978-1-4615-0067-4_32

[12] Jolivet R, Magistretti PJ, Weber B (2009) Deciphering neuron-glia compartmentalization in cortical energy metabolism. Front Neuroenergetics doi:10.3389/neuro.14.004.2009 10.3389/neuro.14.004.2009 PMC2715922 19636395

[13] Khatri N, Man HY (2013) Synaptic Activity and Bioenergy Homeostasis: Implications in Brain Trauma and Neurodegenerative Diseases. Front Neurol. 4: : 199. eCollection 2013. 10.3389/fneur.2013.00199 PMC3858785 24376435

[14] Nomura A, Shigemoto R, Nakamura Y, Okamoto N, Mizuno N, et al (1994) Developmentally regulated postsynaptic localization of a metabotropic glutamate receptor in rat rod bipolar cells. Cell 77: 361–9. 8181056 10.1016/0092-8674(94)90151-1

[15] Dhingra A, Lyubarsky A, Jiang M, Pugh EN Jr, Birnbaumer L, et al (2000) The light response of ON bipolar neurons requires G[alpha]o. J Neurosci 20: 9053–9058. 11124982 10.1523/JNEUROSCI.20-24-09053.2000 PMC6773027

[16] Imai S, Armstrong CM, Kaeberlein M, Guarente L (2000) Transcriptional silencing and longevity protein Sir2 is an NAD-dependent histone deacetylase. Nature 403: 795–800. 10693811 10.1038/35001622

[17] Brunet A, Sweeney LB, Sturgill JF, Chua KF, Greer PL, et al (2004) Stress-dependent regulation of FOXO transcription factors by the SIRT1 deacetylase. Science 303: 2011–2015. 14976264 10.1126/science.1094637

[18] Kaeberlein M, McVey M, Guarente L (1999) The SIR2/3/4 complex and SIR2 alone promote longevity in *Saccharomyces cerevisiae* by two different mechanisms. Genes Dev. 13: 2570–2580. 10521401 10.1101/gad.13.19.2570 PMC317077

[19] Mostoslavsky R, Chua KF, Lombard DB, Pang WW, Fischer MR, et al (2006) Genomic instability and aging-like phenotype in the absence of mammalian SIRT6. Cell 124: 315–29. 16439206 10.1016/j.cell.2005.11.044

[20] Zhong L, D'Urso A, Toiber D, Sebastian C, Henry RE, et al (2010) The histone deacetylase Sirt6 regulates glucose homeostasis via Hif1alpha. Cell 140: 280–93. 20141841 10.1016/j.cell.2009.12.041 PMC2821045

[21] Cheng HL, Mostoslavsky R, Saito S, Manis JP, Gu Y, et al (2003) Developmental defects and p53 hyperacetylation in Sir2 homolog (SIRT1)-deficient mice. Proc. Natl. Acad. Sci USA 100: 10794–10799. 12960381 10.1073/pnas.1934713100 PMC196882

[22] Fernandez DC, Pasquini LA, Dorfman D, Aldana Marcos HJ, Rosenstein RE (2012) Ischemic conditioning protects from axoglial alterations of the optic pathway induced by experimental diabetes in rats. PLoSOne 7(12): e51966 doi:10.1371/journal.pone.0051966 10.1371/journal.pone.0051966 PMC3527393 23284834

[23] Hennig AK, Peng G, Chen S (2013) Transcription Coactivators p300 and CBP Are Necessary for Photoreceptor-Specific Chromatin Organization and Gene Expression. PLoS One 10.1371/journal.pone.0069721 10.1371/journal.pone.0069721 PMC3724885 23922782

[24] Izumi Y, Shimamoto K, Benz AM, Hammerman SB, Olney JW, et al (2002) Glutamate transporters and retinal excitotoxicity. Glia 39: 58–68. 12112376 10.1002/glia.10082

[25] Ban N, Ozawa Y, Inaba T, Miyake S, Watanabe M, et al (2013) Light-dark condition regulates sirtuin mRNA levels in the retina. Exp Gerontol 48: 1212–7. 23648587 10.1016/j.exger.2013.04.010

[26] - Howarth C, Gleeson P, Attwell D (2012) Updated energy budgets for neural computation in the neocortex and cerebellum. J Cereb Blood Flow Metab 32: 1222–32 doi:10.1038/jcbfm.2012.35 22434069 10.1038/jcbfm.2012.35 PMC3390818

[27] Dryja TP, McGee TL, Berson EL, Fishman GA, Sandberg MA, et al (2005) Night blindness and abnormal cone electroretinogram ON responses in patients with mutations in the GRM6 gene encoding mGluR6. Proc Natl Acad Sci USA 102: 4884–9. 15781871 10.1073/pnas.0501233102 PMC555731

[28] Sebastián C, Satterstrom FK, Haigis MC, Mostoslavsky R (2012) From Sirtuin Biology to Human Diseases: An Update. J Biol Chem 287: 42444–52. 23086954 10.1074/jbc.R112.402768 PMC3522245

[29] Zhong Q, Kowluru RA (2011) Epigenetic changes in mitochondrial superoxide dismutase in the retina and the development of diabetic retinopathy. Diabetes 60: 1304–1313. 21357467 10.2337/db10-0133 PMC3064104

[30] Pelzel HR, Schlamp CL, Waclawski M, Shaw MK, Nickells RW (2012) Silencing of Fem1cR3 gene expression in the DBA/2J mouse precedes retinal ganglion cell death and is associated with histone deacetylase activity. Invest Ophthalmol Vis Sci 53: 1428–1435. 22297488 10.1167/iovs.11-8872 PMC3339913

[31] Wei L, Liu B, Tuo J, Shen D, Chen P, et al (2012) Hypomethylation of the IL17RC Promoter Associates with Age-Related Macular Degeneration. Cell Rep 2: 1151–8. 23177625 10.1016/j.celrep.2012.10.013 PMC3513594

[32] Yoo CB, Jones PA (2006) Epigenetic therapy of cancer: past, present and future. Nat Rev Drug Discov 5: 37–50. 16485345 10.1038/nrd1930

