# Supplementary figures and images for: SIRT6 Is Required for Normal Retinal Function
Source: PLoS One. 2014 Jun 4;9(6):e98831. doi: 10.1371/journal.pone.0098831 (PMC4045872; doi:10.1371/journal.pone.0098831)

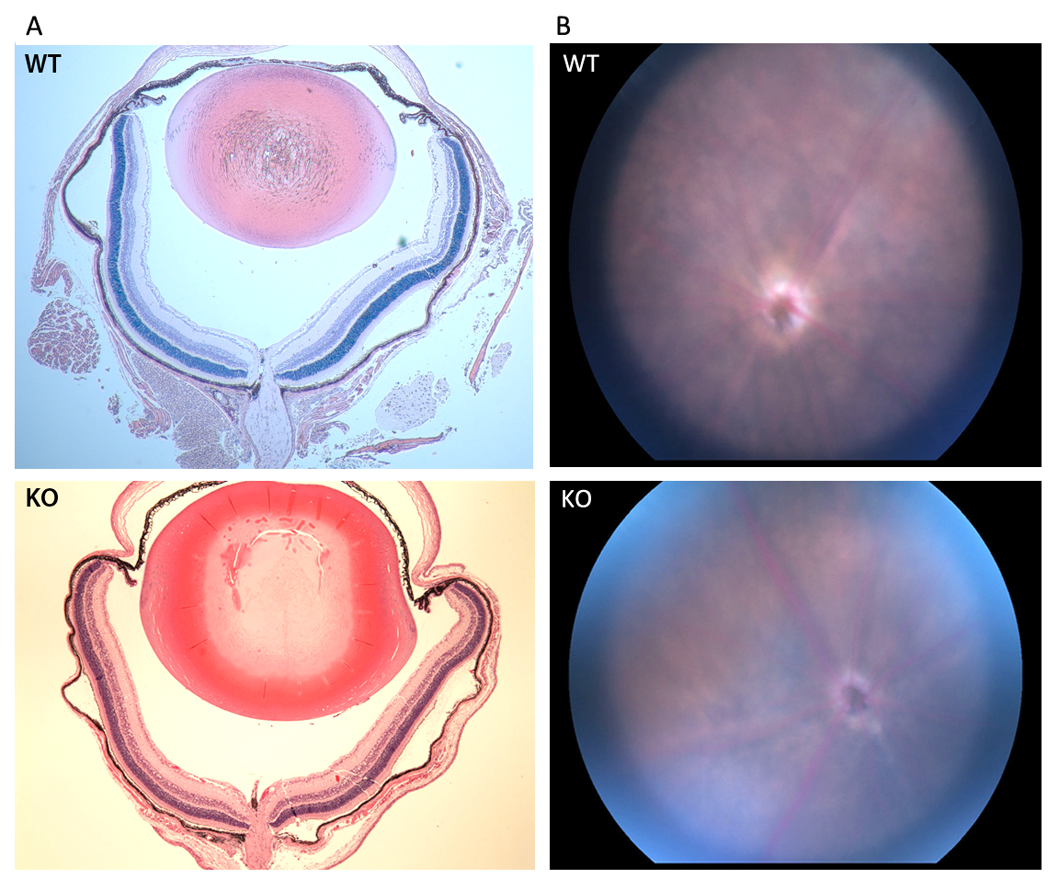

Supplement: Figure S1 — Histological analysis and eye fundus. a) Representative low magnification (4X) cross-sections of eyes from WT and SIRT6KO mice. General structure, size and optic nerve head can be observed. No distinctive alteration is detected in SIRT6KO retinas b) Representative fundus images were taken from WT and 2-week-old mouse using the endoscopic fundus imaging system. Posterior pole of the fundus can be observed. (TIF) [file pone.0098831.s001.tif]

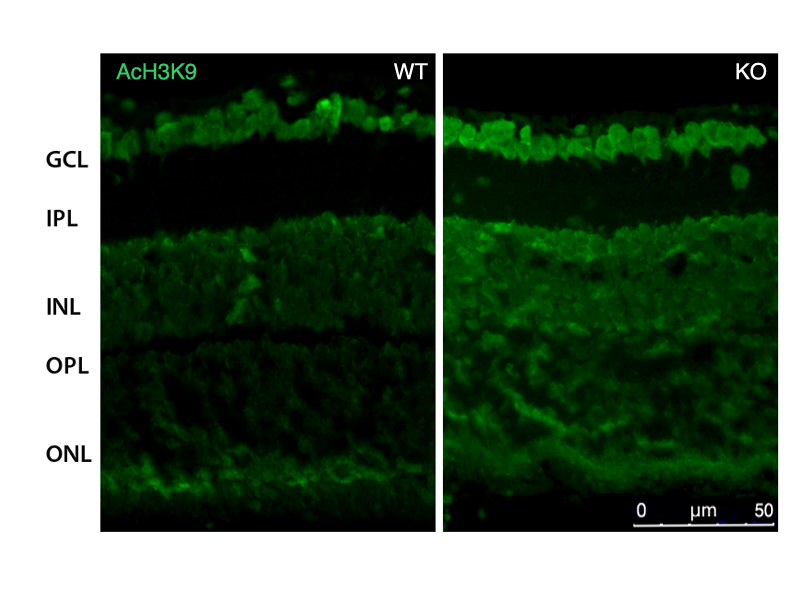

Supplement: Figure S2 — H3K9 acetylation is shown by immunofluoescence. Ganglion Cell Layer (GCL), Inner Plexiform Layer (IPL), Inner nuclear Layer (INL) Outer Plexiform Layer (OPL), Outer Nuclear Layer (ONL), Retinal Pigment Epithelium (RPE). (TIF) [file pone.0098831.s002.tif]
