# Supplementary material for: SIRT6 Is Required for Normal Retinal Function
Source: PLoS One. 2014 Jun 4;9(6):e98831. doi: 10.1371/journal.pone.0098831 (PMC4045872; doi:10.1371/journal.pone.0098831)
Supplement: File S1 — Supporting Methodology. (DOCX) [file pone.0098831.s003.docx]

Supporting Methodology

**Eye fundus**

Eyes were examined for clinical status using a binocular fundus microscope with coaxial illumination. A drop of sterile physiological solution was placed on the cornea and a microscope coverslip served as a lens to equalize refraction. Eyes were examined for engorged blood vessels, constricted blood vessels (“cuffing”), white linear lesions, subretinal hemorrhages, and retinal detachment. The fundus was imaged using the Micron II small animal retinal imaging AD camera (Phoenix Research Laboratories, INC).
